# Identification and Verification of Feature Immune-Related Genes in Patients With Hypertrophic Cardiomyopathy Based on Bioinformatics Analyses

**DOI:** 10.3389/fcvm.2021.752559

**Published:** 2021-10-26

**Authors:** Xifeng Zheng, Guangyan Liu, Ruina Huang

**Affiliations:** ^1^Department of Geriatrics, Affiliated Hospital of Guangdong Medical University, Zhanjiang, China; ^2^Department of Cardiology, Affiliated Hospital of Guangdong Medical University, Zhanjiang, China

**Keywords:** biomarkers, hypertrophic cardiomyopathy, immune-related gene, diagnosis model, random forest—ensemble classifier, LASSO

## Abstract

**Objective:** To identify feature immune-related genes (IRGs) in patients with hypertrophic cardiomyopathy (HCM) and verify their ability to diagnose HCM.

**Methods:** The GSE160997 dataset on cardiac tissue from 18 HCM patients and 5 controls was downloaded from the Gene Expression Omnibus database. A false discovery rate <0.05 and |log2 fold change| >1 were the filters applied to identify the differentially expressed genes (DEGs). The differentially expressed IRGs were the intersection results between the DEGs and an IRG dataset from the IMMPORT database. The protein-protein interaction network of differentially expressed IRGs was constructed, and the top 20 hub genes with the most adjacent nodes in the network were selected. The least absolute shrinkage and selection operator regression algorithm and a random forest algorithm were used to identify the feature IRGs as biomarkers that were then verified against GSE36961.

**Results:** A total of 1079 DEGs were identified in GSE160997. Gene Ontology and Kyoto Encyclopedia of Genes and Genomes pathway enrichment analyses indicated that immune-related mechanisms play an important role in the pathogenesis of HCM. A total of 121 differentially expressed IRGs were identified, and 5 feature IRGs were selected, 4 of which were confirmed as potential biomarkers of HCM by external verification with excellent discrimination ability. A diagnosis model of HCM based on the four feature IRGs was developed and visualized as a nomogram with a C-index of 0.925 (95% confidence interval 0.869–0.981).

**Conclusion:** Our study identified four feature IRGs as biomarkers for the diagnosis of HCM, offering an innovative perspective of the underlying immune-related pathological molecular mechanisms.

## Introduction

Hypertrophic cardiomyopathy (HCM) is a hereditary heart disease that is defined as unexplained, isolated, progressive myocardial hypertrophy, usually accompanied by heart failure or arrhythmia. It is considered the primary underlying condition in sudden cardiac death in young adults and athletes. Mutations in the genes encoding proteins of the cardiomyocytic contractile apparatus are considered the main cause of HCM. However, the relationship between sarcomere mutations and clinical outcomes remains unpredictable in patients with HCM, largely because of genetic and phenotypic heterogeneity (1, 2). During the past two decades, the continuous improvement of gene sequencing technology has enabled the diagnosis of HCM among populations with a family history and in asymptomatic patients through genetic testing to avoid sudden death. This has become a class I recommendation for supplementary testing according to the European Society of Cardiology and American Heart Association/American College of Cardiology guidelines (3, 4).

Sarcomere protein gene (such as MYBPC3, MYH7, TNNT2, TNNI3, TPM1, MYL2, MYL3, and ACTC) mutations have become targets in the genetic testing of HCM. Unfortunately, in the genetic testing of different HCM cohorts, up to 50–68% of patients were negative for sarcomere protein gene mutations (5). The signaling pathways and regulatory networks underlying the pathogenesis of HCM remain unclear to date. Thus, a systematic analysis of its transcriptional map might provide useful information about the overall underlying molecular mechanisms and pathological processes of HCM and aid in the diagnosis and treatment of HCM.

**Figure 1:**
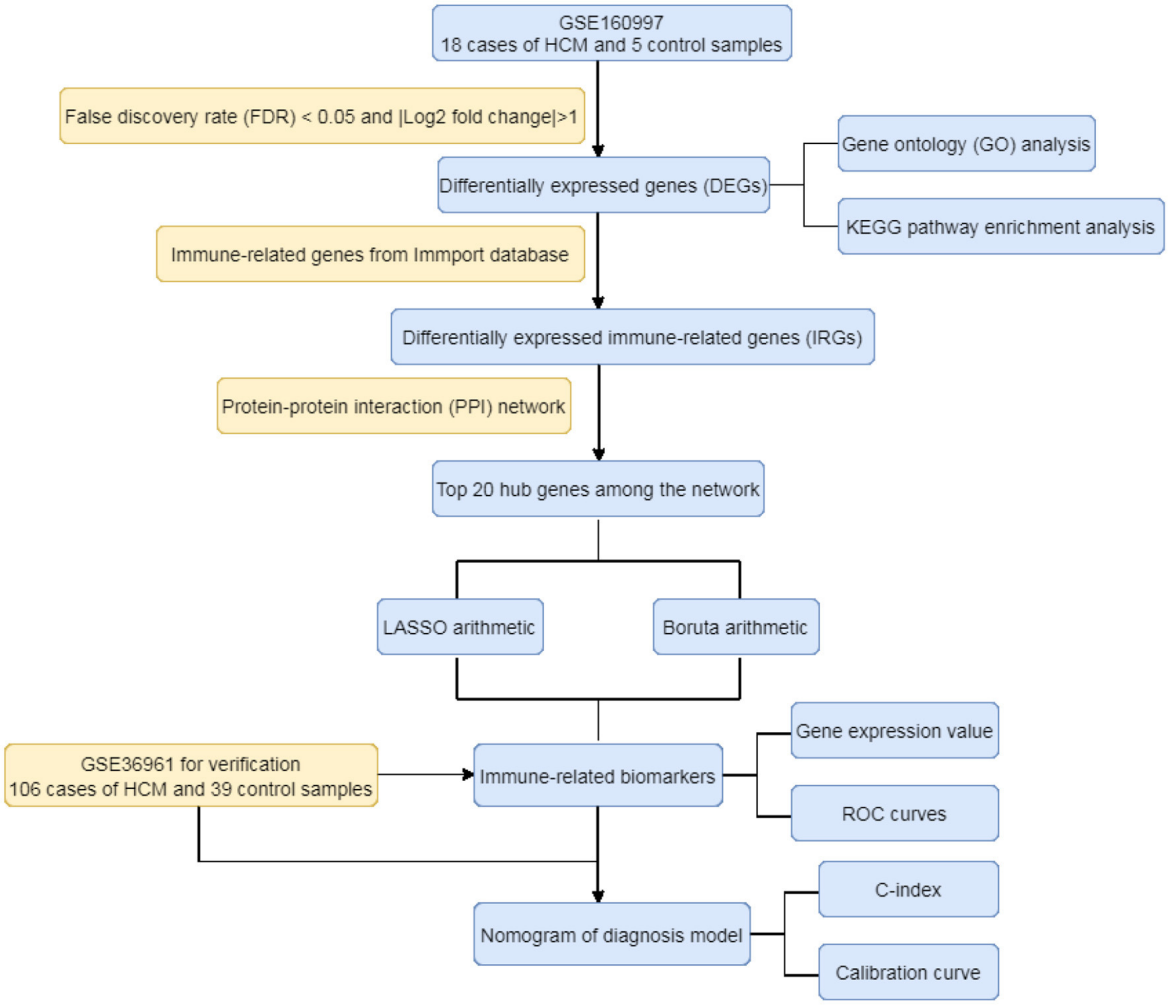
The flowchart of the study.

As the flowchart showed in Figure 1, this study aimed to explore the high-throughput sequencing gene expression data of HCM patients and normal samples, construct the differential gene expression profile of HCM, annotate the gene ontology and potential signal pathways, and identify valuable biomarkers that may further the gene test diagnosis of HCM.

## Materials and Methods

### Data Source and Differentially Expressed Gene Analyses

There are five comparatively valuable datasets that contain more than 10 samples of HCM in GEO database, namely GSE160997 (6), GSE143786 (7), GSE148602 (8), GSE130036 (9), and GSE36961. Among these five datasets, GSE160997, GSE130036, and GSE36961 focus on transcriptome information and a previous study of our team had explored GSE130036 (10). The high-throughput sequencing dataset GSE160997 used in this study contained data regarding myocardial tissue of 18 HCM patients and 5 healthy controls. All data were statistically analyzed and visualized using R software (version 4.0.3) and its relevant packages. The original data of all samples were normalized and differentially expressed genes (DEGs) were identified using the following filters: a false discovery rate [FDR (11)] <0.05 and |Log2 fold change| >1 analyzed with the limma package (12). The Gene Ontology (GO) and Kyoto Encyclopedia of Genes and Genomes (KEGG) pathway enrichment analyses of DEGs were further explored by the “ClusterProfiler” (13) and “org.Hs.eg.db” (14) packages. The results were visualized with the “ggplot2” (15) package.

### Immune-Related Gene Analyses

A dataset with 1,793 unique immune-related genes (IRGs) that had been confirmed in previous studies as being involved in the natural immune process was downloaded from the IMMPORT database [(16); https://www.immport.org/shared/]. The differentially expressed IRGs were the intersection results between the DEGs and the 1793 IRGs. The protein-protein interaction (PPI) network of differentially expressed IRGs was constructed using the STRING database [(17); https://string-db.org/, Version:11.0], with the highest confidence level defined as an interaction score of more than 0.9. Subsequently, the top 20 hub genes with the most adjacent nodes in the PPI network were selected.

### Feature IRGs Identified and Correlation Analysis

The least absolute shrinkage and selection operator (LASSO) regression arithmetic and Boruta arithmetic (18) were used independently to identify the feature IRGs from the 20 hub genes. The former is a regression analysis algorithm that uses regularization to improve prediction accuracy (19). The latter is a random forest method that can provide a numerical estimate of feature importance (20). In addition, the R “corrplot” package (21) was used to analyze a potential correlation among the selected feature IRGs.

### Feature IRGs Verification and Diagnosis Model Constructed

The selected feature IRGs were further verified using the independent dataset GSE36961 with 106 HCM patients and 39 healthy controls. The gene expression values were displayed by boxplots, and receiver-operating characteristic (ROC) curves were used to assess the discrimination ability of the feature IRGs. A five-fold cross-validation was conducted 500 times on the GSE36961 cohort to conform the reliability of the area under the ROC curves (AUC) of the selected biomarkers. Moreover, an online webtool NetworkAnalyst (22) was used to further explore the potential miRNA—feature IRG regulatory network and transcription factor (TF)—feature IRG regulation mechanisms. Finally, a diagnosis model combining the selected biomarkers was developed by logistic regression and visualized as a nomogram (23). The C-statistic and calibration curves were used to evaluate the identification ability and calibration of the model.

## Results

### DEGs Between HCM and Normal Samples

**Figure 2:**
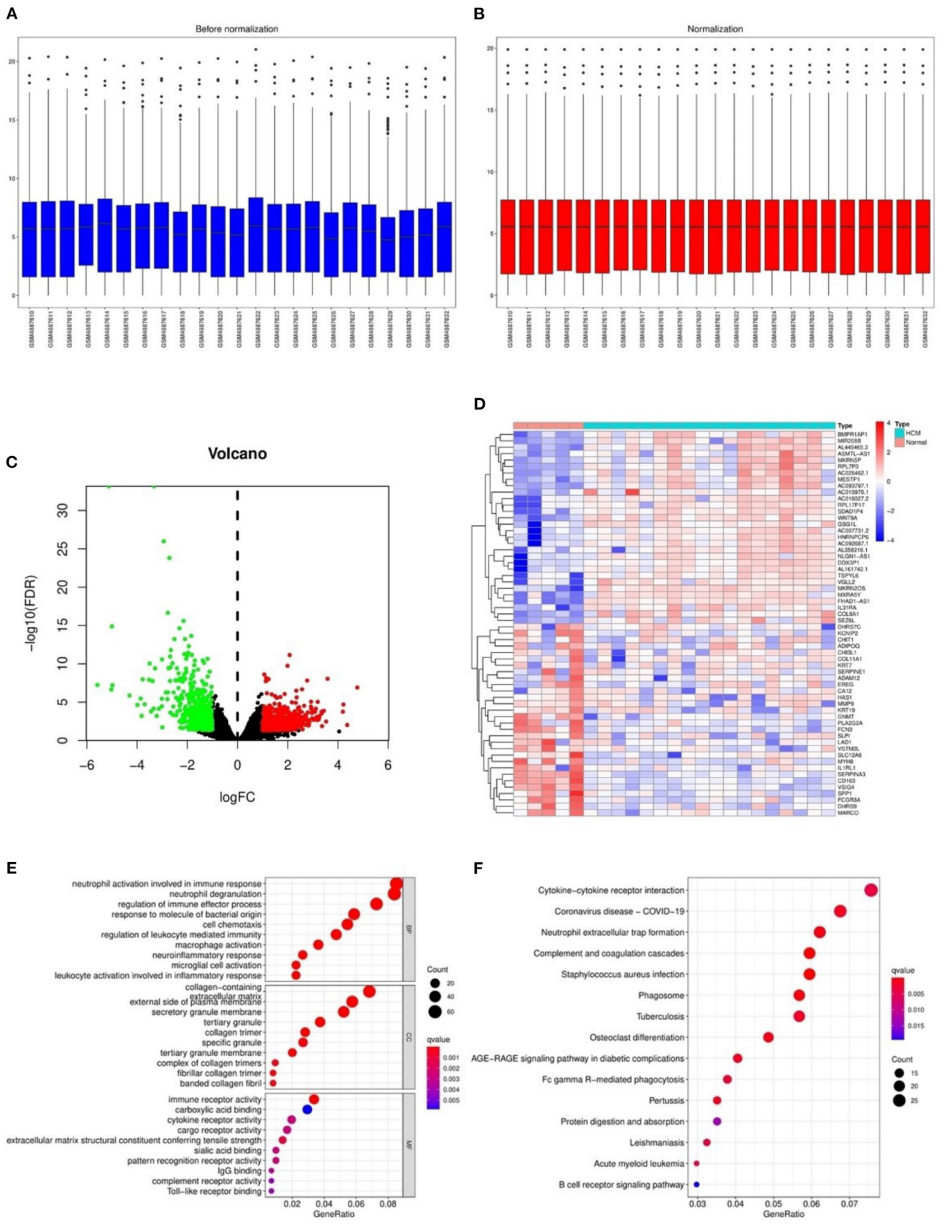
The differentially expressed genes in GSE160997 and the Gene Ontology annotation and Kyoto Encyclopedia of Genes and Genomes pathway enrichment analysis. **(A)** The boxplot of GSE160997 before normalization. **(B)** The boxplot of GSE160997 after normalization. **(C)** The Volcano plot of 1,079 DEGs, with 618 significantly upregulated (red) and 461 significantly downregulated (green). **(D)** The heatmap of top 30 DEGs. **(E)** The gene ontology (GO) annotation of DEGs. **(F)** The Kyoto Encyclopedia of Genes and Genomes (KEGG) enrichment analysis of DEGs.

After the normalization of GSE160997 dataset (Figures 2A,B), a total of 1,079 DEGs were identified in the GSE160997 dataset, and filtering resulted in 618 significantly upregulated and 461 significantly downregulated DEGs (Figure 2C). The top 30 DEGs were displayed in a heatmap (Figure 2D). The GO annotation and KEGG pathway enrichment analyses of DEGs are illustrated in Figures 2E,F. It became obvious that inflammation and immune-related signaling pathways and biological regulatory functions played a dominant role in the enrichment results of the DEGs, which prompted us to further explore the IRGs among the DEGs.

### The Information of Immune-Related Genes

**Figure 3:**
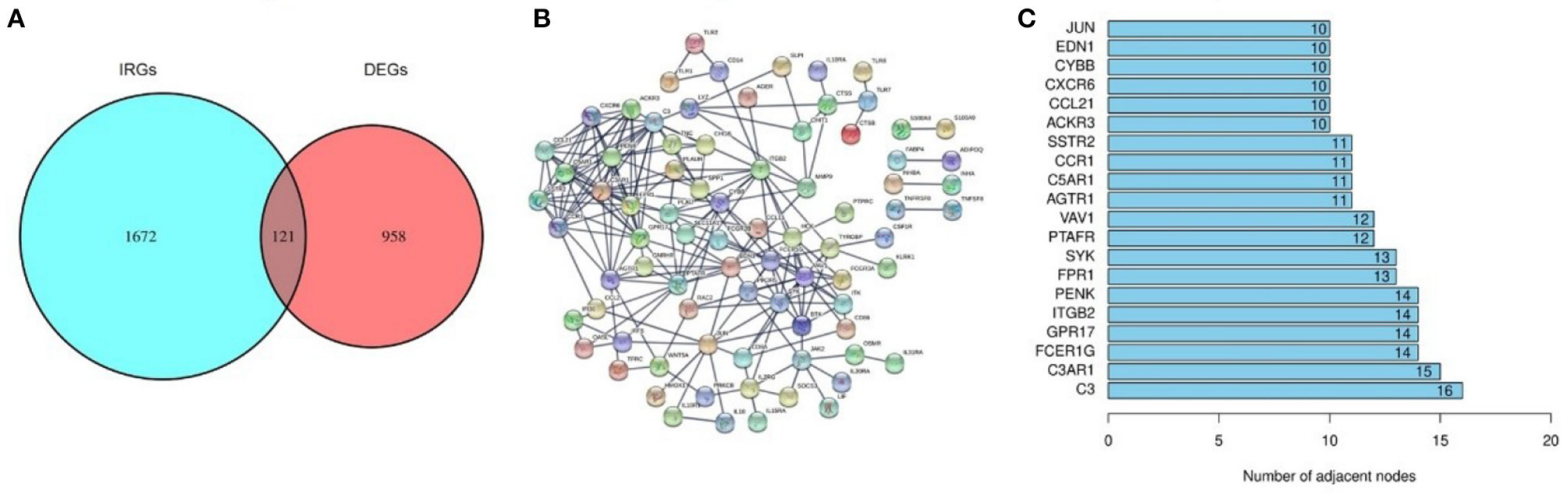
The differentially expressed Immune-related genes and PPI network. **(A)** The intersection result of DEGs and IRGs. **(B)** The protein-protein interaction (PPI) network of 121 differentially expressed IRGs. **(C)** The top 20 genes with most adjacent nodes among the PPI network.

A total of 121 differentially expressed IRGs between the DEGs and an IRG dataset from the IMMPORT database were identified (Figure 3A). These 121 differentially expressed IRGs formed a close PPI network interaction at a confidence score >0.9 (Figure 3B). The top 20 hub genes with the most adjacent nodes in the PPI network are listed in Figure 3C, and the detailed information is listed in Table 1.

**Table 1:** The detail information of the top-20 immune-related genes with most adjacent nodes in protein-protein interaction network.

**Gene symbol**	**logFC**	**FDR**	**Adjacent** **nodes**	**Description**
C3	−1.99	<0.001	16	Complement C3. Also known as: AHUS5, ARMD9, ASP, C3a, C3b, CPAMD1, and HEL-S-62p
C3AR1	−2.09	<0.001	15	Complement C3a receptor 1. Also known as: AZ3B, C3AR, and HNFAG09
FCER1G	−2.10	<0.001	14	Fc fragment of IgE receptor Ig. Also known as: FCRG
ITGB2	−1.96	<0.001	14	Integrin subunit beta 2. Also known as: CD18, LAD, LCAMB, LFA-1, MAC-1, MF17, and MFI7
GPR17	2.32	0.005	14	G protein-coupled receptor 17
PENK	2.27	0.009	14	Proenkephalin. Also known as: PE and PENK-A
SYK	−1.41	<0.001	13	Spleen associated tyrosine kinase. Also known as: IMD82 and p72-Syk
FPR1	−2.24	<0.001	13	Formyl peptide receptor 1. Also known as: FMLP and FPR
VAV1	−1.60	<0.001	12	Vav guanine nucleotide exchange factor 1. Also known as: VAV
PTAFR	−1.25	0.003	12	Platelet activating factor receptor. Also known as: PAFR
AGTR1	−1.23	0.008	11	Angiotensin II receptor type 1. Also known as: AG2S, AGTR1B, AT1, AT1AR, AT1B, AT1BR, AT1R, AT2R1, and HAT1R
C5AR1	−1.59	<0.001	11	Complement C5a receptor 1. Also known as: C5A, C5AR, C5R1, and CD88
CCR1	−2.65	<0.001	11	C-C motif chemokine receptor 1. Also known as: CD191, CKR-1, CKR1, CMKBR1, HM145, MIP1aR, and SCYAR1
SSTR2	−1.39	<0.001	11	Somatostatin receptor 2
CYBB	−1.84	<0.001	10	Cytochrome b-245 beta chain. Also known as: AMCBX2, CGD, CGDX, GP91-1, GP91-PHOX, GP91PHOX, IMD34, NOX2, and p91-PHOX
CCL21	−2.18	0.004	10	C-C motif chemokine ligand 21. Also known as: 6Ckine, CKb9, ECL, SCYA21, SLC, and TCA4
EDN1	−1.31	0.003	10	Endothelin 1. Also known as: ARCND3, ET1, HDLCQ7, PPET1, and QME
ACKR3	1.17	<0.001	10	Atypical chemokine receptor 3. Also known as: CMKOR1, CXC-R7, CXCR-7, CXCR7, GPR159, RDC-1, and RDC1
JUN	1.18	<0.001	10	Jun proto-oncogene, AP-1 transcription factor subunit. Also known as: AP-1, AP1, c-Jun, cJUN, and p39
CXCR6	1.73	0.002	10	C-X-C motif chemokine receptor 6. Also known as: BONZO, CD186, CDw186, STRL33, and TYMSTR

### Feature IRGs Identified and Correlation Analysis

**Figure 4:**
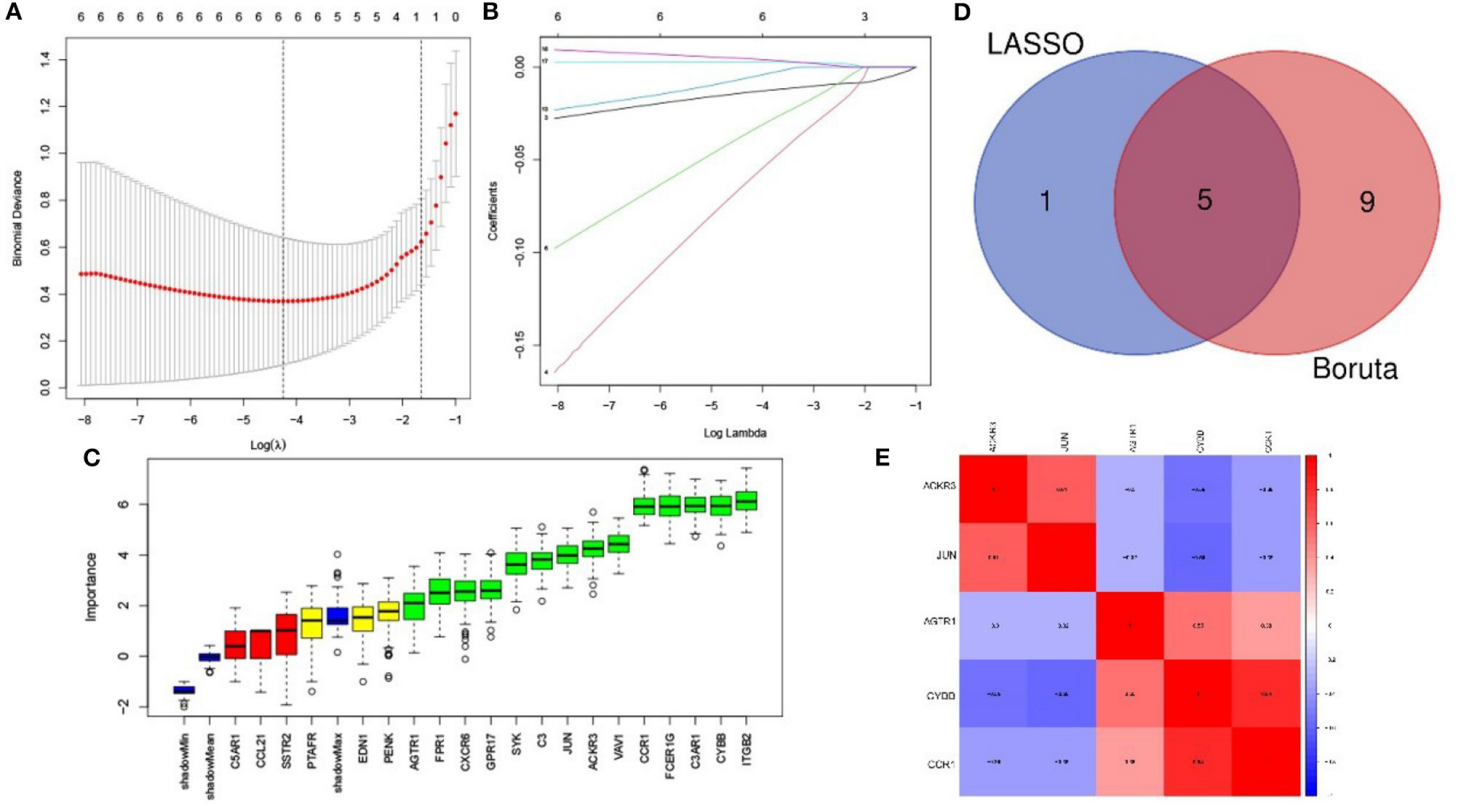
Feature immune-related genes identified and correlation analysis. **(A)** The optimal lambda selection by LASSO. **(B)** The coefficient profiles of the six hub genes. **(C)** Feature IRGs selected by Boruta arithmetic. Genes in green are regarded as important feature IRGs while neutral in yellow and are rejected in red according to the score of feature importance. **(D)** The intersection result of LASSO and Boruta arithmetic. **(E)** The correlation analysis of the feature IRGs.

The LASSO arithmetic selected 6 feature IRGs (Figures 4A,B) and the Boruta arithmetic 14 feature IRGs (Figure 4C). Five feature IRGs (ACKR3, AGTR1, CCR1, CYBB, and JUN) were identified by both methods (Figure 4D). The correlation analysis result displaying the Pearson correlation values indicated that there was a strong positive correlation between CYBB and CCR1 and a moderate positive correlation between ACKR3 and JUN (Figure 4E).

### Feature IRG Verification and Diagnosis Model Constructed

**Figure 5:**
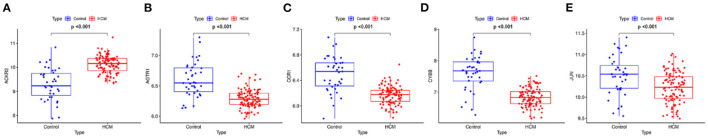
Gene expression of the five-feature immune-related genes. The gene expression value of the five feature IRGs. **(A)** Boxplot of ACKR3 in GSE36961. **(B)** Boxplot of AGTR1 in GSE36961. **(C)** Boxplot of CCR1 in GSE36961. **(D)** Boxplot of CYBB in GSE36961. **(E)** Boxplot of JUN in GSE36961.

**Figure 6:**
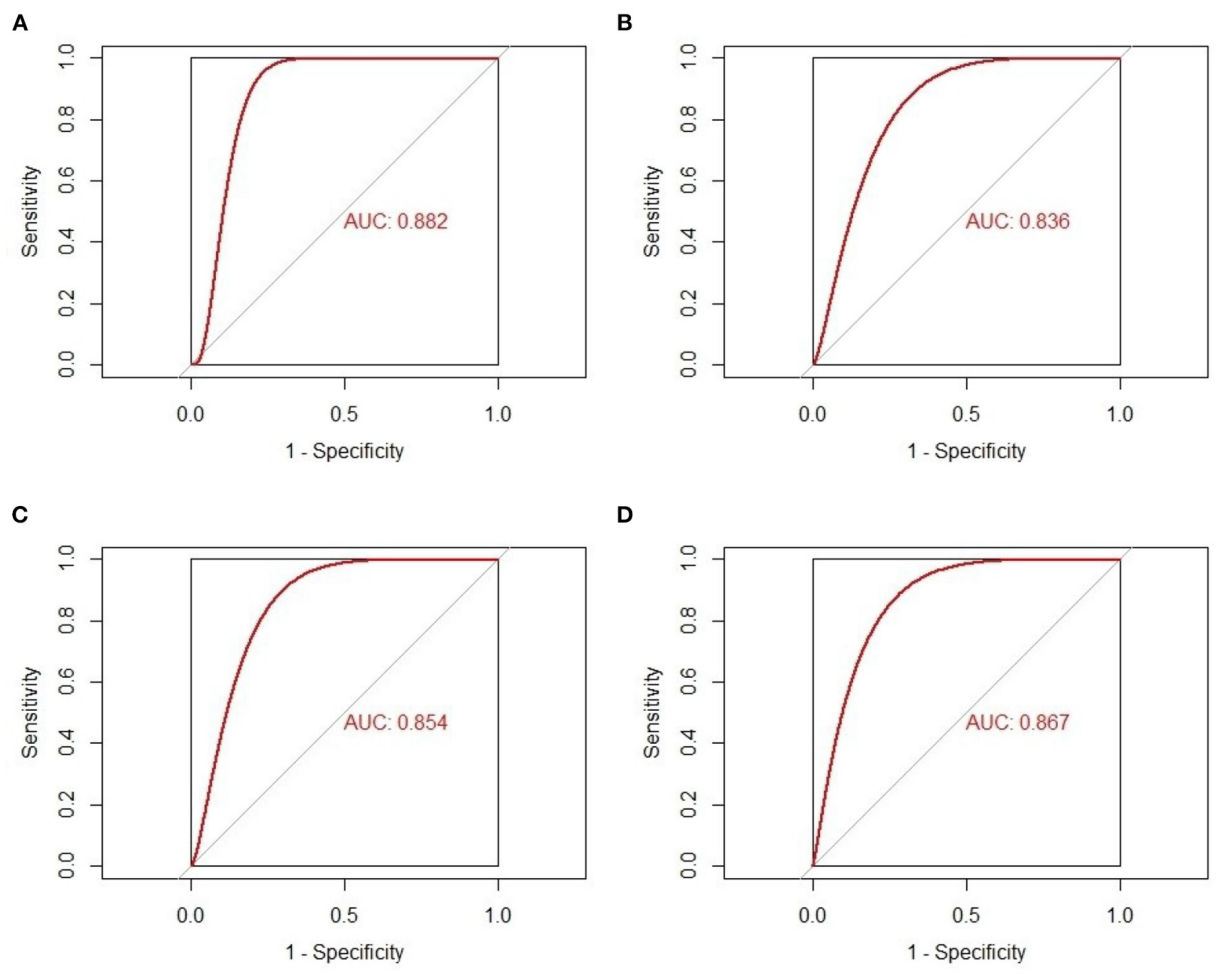
The receiver-operating characteristic curves of the four-feature immune-related genes as biomarkers of hypertrophic cardiomyopathy. **(A)** AUC of CYBB in GSE36961. **(B)** AUC of AGTR1 in GSE36961. **(C)** AUC of CCR1 in GSE36961. **(D)** AUC of ACKR3 in GSE36961.

The gene expression values of the five selected IRGs in GSE36961 are illustrated in Figure 5, and the original data are presented in the Supplementary File. It is worth noting that JUN was highly expressed in the normal controls in GSE36961, which was contrary to the previous result. The gene expressions of the remaining four IRGs (ACKR3, AGTR1, CCR1, and CYBB) were consistent with the previous analysis and were statistically significant, suggesting them as potential diagnostic biomarkers of HCM. The AUC of the four biomarkers were all remarkable, indicating their ability to contribute to the diagnosis of HCM (Figure 6). The results of 500 times five-fold cross-validation show that the average AUC scores of CYBB, CCR1, AGTR1 and ACKR3 were 0.883, 0.856, 0.842, and 0.865 respectively.

**Figure 7:**
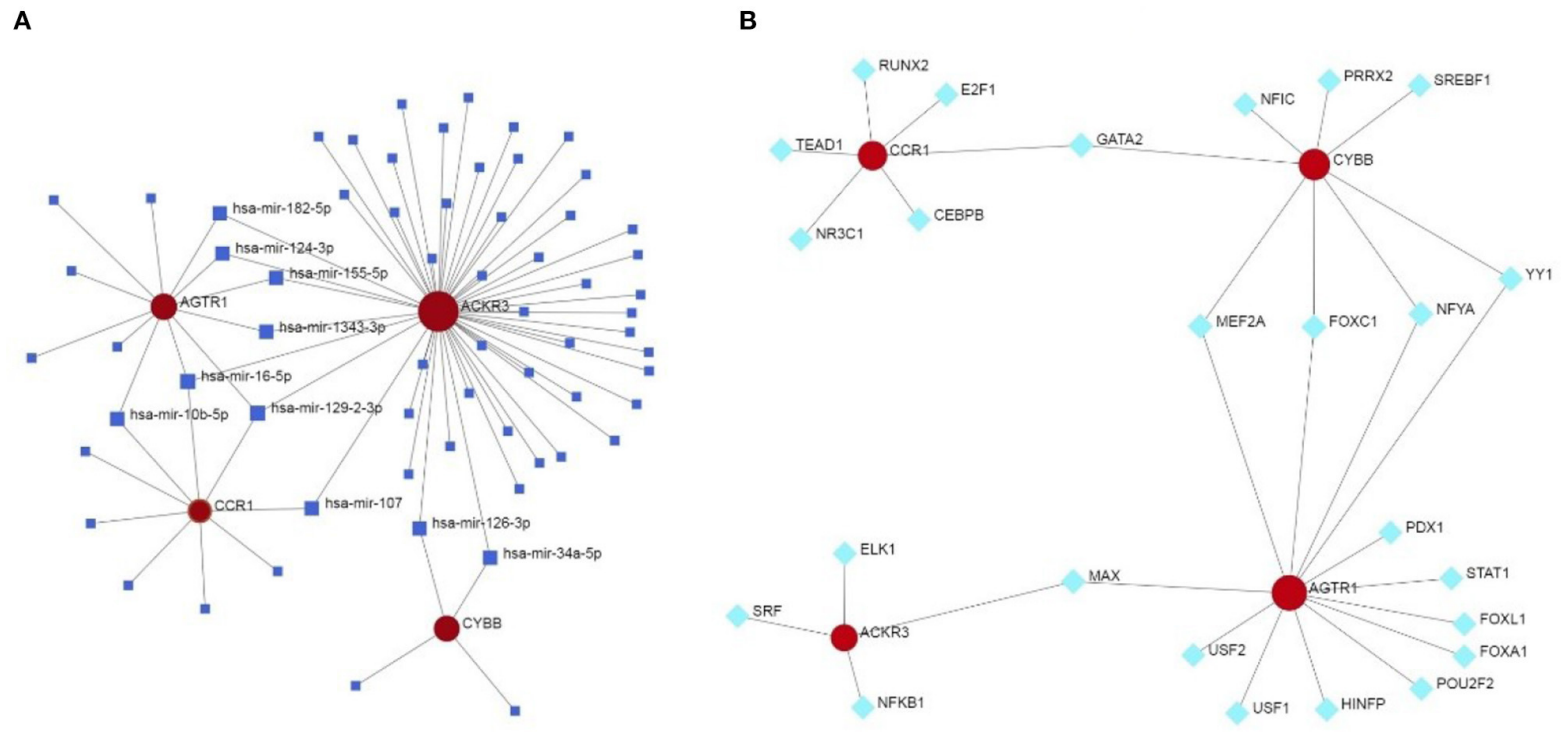
The miRNA—feature IRG regulatory network and transcription factor (TF)—feature IRG regulatory network. **(A)** The miRNA—feature IRG regulatory network. **(B)** The transcription factor (TF)—feature IRG regulatory network.

**Figure 8:**
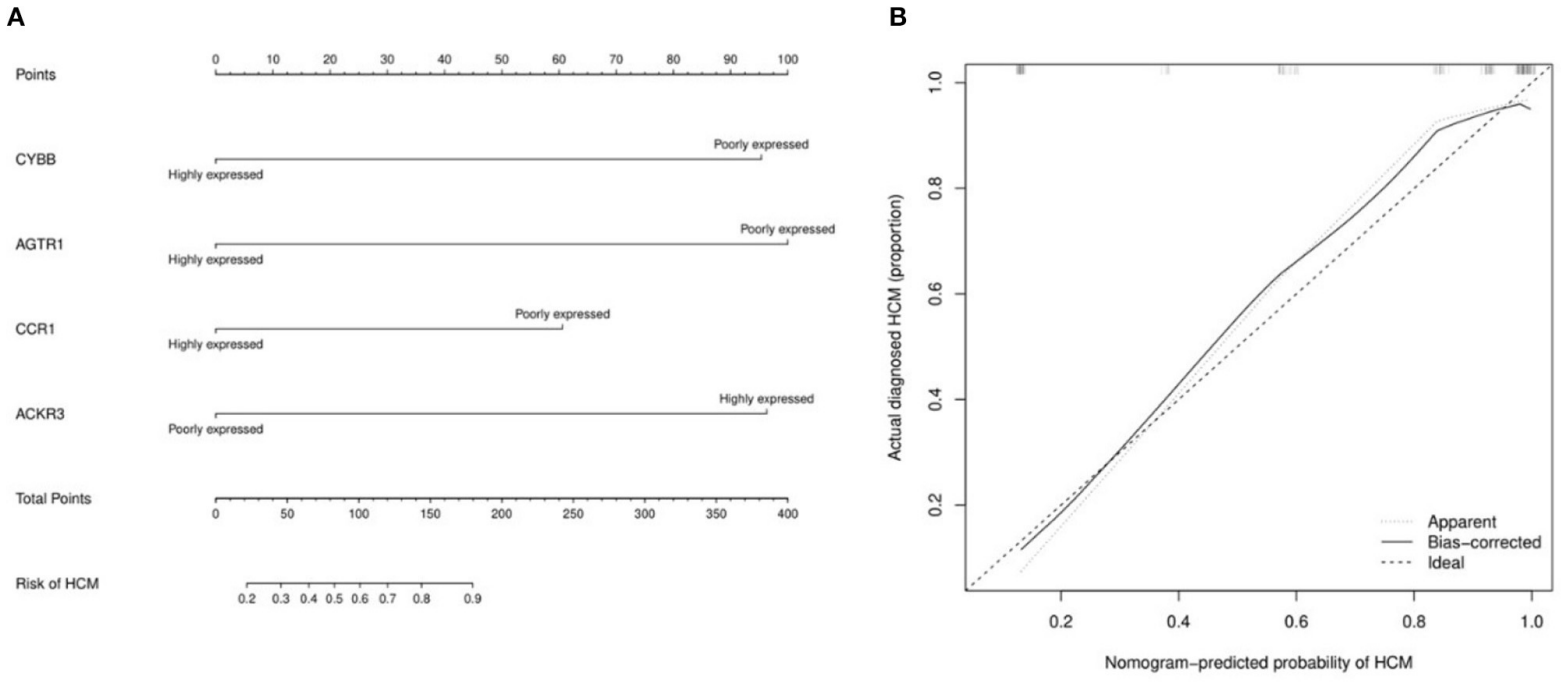
The nomogram and calibration curve of the diagnosis model for hypertrophic cardiomyopathy based on four feature immune-related genes. **(A)** Nomogram for the diagnosis model of HCM. The observed value of each feature gene was assigned a certain number of points by drawing a vertical line toward the top points scale. The sum of the points for each gene corresponded to the individual risk of HCM. **(B)** Calibration curve for the diagnosis model of HCM.

The miRNA—feature IRG regulatory network and transcription factor (TF)—feature IRG regulatory network were listed as Figure 7. The four IRGs were transformed into binary variables according to the median value of gene expression, based on which a diagnosis model was constructed by logistic regression that was visualized as a nomogram (Figure 8A). The C-index of the diagnosis model was 0.925 (95% confidence interval 0.869–0.981) with appropriate calibration (Figure 8B).

## Discussion

HCM is a common inherited cardiovascular disease that occurs in one of 500 people in the general population and leads to heart failure, fatal arrhythmia, and stroke. Mutations in genes encoding proteins of the cardiomyocytic contractile apparatus are considered the main cause of HCM. Genetic tests focusing on sarcomere protein genes have been recommended in current guidelines and experience widespread adoption. However, with the accumulation of experimental data (24–26), approximately two-thirds of HCM patients were shown not to have sarcomere protein mutations. Therefore, it is necessary to explore alternative molecular mechanisms and identify more valuable biomarkers of HCM by utilizing contemporary methods to analyze biological complexity with an emphasis on network medicine (27).

According to bioinformatics analysis, the GO annotation and KEGG enrichment analysis of the DEGs in GSE160997 were dominated by immune and inflammatory response mechanisms, such as neutrophil and macrophage activation, neutrophil degranulation, regulation of leukocyte-mediated immunity, and immune effector processes. Currently, inflammation and immunity attract substantial interest with regard to their role in the pathophysiology of cardiovascular diseases. Some previous studies (28–31) suggested that immune and inflammatory response mechanisms may play an important role in myocardial hypertrophy and remodeling. In contrast, most research has focused on heart failure and myocardial remodeling after acute myocardial infarction, with extremely limited evidence on HCM.

The preliminary results of bioinformatics analysis enabled further exploration of IRGs as biomarkers of HCM. A dataset with 1,793 unique IRGs was downloaded from the IMMPORT database and used to identify the IRGs among the DEGs in GSE160997. A total of 121 IGRs were selected from the 1,079 DEGs, and 5 feature IRGs were identified as candidate biomarkers by both the LASSO and Boruta arithmetic. Finally, four feature IRGs were confirmed as biomarkers with an excellent AUC score using the GSE36961 dataset of HCM patients and 39 healthy controls. Our novel diagnosis model of HCM constructed based on these four feature IRGs showed high discrimination ability (C-index = 0.925) and favorable calibration. This pioneering research offers a new perspective on the identification of potential biomarkers that might aid in the diagnosis of HCM.

Angiotensin II receptor type 1 (AGTR1) is a significant effector of the renin-angiotensin-aldosterone system, which is associated with cardiovascular diseases, especially cardiac remodeling. The system consists of two regulatory pathways: a pathological pro-inflammatory pathway and a protective anti-inflammatory pathway. AGTR1, involved in the former, can promote vasoconstriction, cell proliferation, inflammation, oxidative stress, hypertrophy, and fibrosis, and angiotensin II–type 1 receptor blockers are widely used to prevent cardiac remodeling after acute myocardial infarction and heart failure (32, 33).

Atypical chemokine receptor 3 (ACKR3) (34), previously known as C-X-C chemokine receptor type 7 (CXCR7), has emerged as a potential hub gene in cardiac development in animal experiments (35–37). Nevertheless, animal studies have focused on the effects of ACKR3 knockout on cardiac development. In this study, the expression of ACKR3 in HCM patients was significantly higher than that in the normal controls in both GSE160997 and GSE36961. At present, no study has explored the effect of a high expression of ACKR3 on cardiac development or structural remodeling, which may be a promising area of research going forward.

CCR1 gene encodes a protein that acts as a chemokine receptor. This protein and its ligands macrophage inflammatory protein 1 (MIP-1, CCL3) and regulated on activation normal T expressed and secreted protein (RANTES, CCL5) can be expressed in monocytes/macrophages/T lymphocytes, whose signaling is the key to recruit effector immune cells to inflammatory sites. Liehn et al. proved that in model mice with myocardial infarction, the deletion of CCR1 can reduce tissue inflammatory injury and protect the myocardium (38). Data from animal and human studies show that CCR1 and its ligands are central mediators of a variety of inflammatory diseases, making the receptor an attractive candidate for therapeutic intervention. At present, CCR1 receptor antagonists are mainly used in clinical trials for the treatment of rheumatoid arthritis (39). Batis et al. selectively blocks the CCR1 receptor through met RANTES therapy, confirming that a treatment strategy based on regulating CCR1/CCR5 mediated cell migration and/or effector function may help limit cardiac tissue damage during chronic Chagas disease (40).

The protein encoded by cytochrome b-245 beta chain (CYBB) gene, also named NOX-2, is a component of cytochrome b-245, which plays an important role in the development of phagocytic microbicidal oxidase system. A consensus has been reached that the deficiency of CYBB leads to decreased NADPH oxidase activity in phagocytes, and neutrophils phagocytize bacteria but do not effectively kill bacteria, and that this might be the potential pathogenesis of chronic granulomatous disease (41). In addition, the gene has also been reported to be associated with ventricular hypertrophy and arrhythmia caused by hypoxia and high-fat diet (42–44).

The strengths of this study are (a) the innovative perspective that immune and inflammation may underlie the molecular mechanism of HCM and (b) the identification of IRGs as diagnostic biomarkers using bioinformatics analysis of transcriptome datasets of HCM patients and healthy controls. Nonetheless, some limitations of this study need to be recognized. First, the study was a retrospective data analysis and lacked detailed clinical or prognostic information, such as the thickness of the ventricular septum and the occurrence of major adverse cardiovascular events in patients with HCM. This limited the further exploration of the relevant genes determining these clinical features or outcomes. Second, although the four feature biomarkers were verified by an independent dataset of sufficient size and achieved excellent discrimination, a larger dataset is essential for verification in the future. Third, the different transcriptome datasets were detected by different sequencing platforms. The baselines of the different transcriptome datasets were inconsistent, indicating that they may only determine whether the expression of characteristic genes is abnormal without the ability to identify a generally applicable reference range.

## Conclusion

The systematic analysis of the transcriptional profiles of HCM and exploration of the potential underlying molecular mechanisms led to the identification of four feature IRGs as biomarkers of HCM. Based on these, a diagnosis model of HCM was developed that brings a novel aspect to the current understanding of the pathological processes and required genetic testing in HCM.

## Data Availability Statement

The datasets presented in this study can be found in online repositories. The names of the repository/repositories and accession number(s) can be found in the article/Supplementary Material.

## Author Contributions

XZ and GL were involved in the conception and design of the study. XZ was responsible for visualization and article writing. RH provided scientific supervision. All authors reviewed and approved the final manuscript.

## Conflict of Interest

The authors declare that the research was conducted in the absence of any commercial or financial relationships that could be construed as a potential conflict of interest.

## Publisher's Note

All claims expressed in this article are solely those of the authors and do not necessarily represent those of their affiliated organizations, or those of the publisher, the editors and the reviewers. Any product that may be evaluated in this article, or claim that may be made by its manufacturer, is not guaranteed or endorsed by the publisher.

## Abbreviations

HCM: hypertrophic cardiomyopathy
GO: gene ontology
KEGG: Kyoto Encyclopedia of Genes and Genomes
DEGs: differentially expressed genes
IRG: immune-related gene
PPI: protein-protein interaction
LASSO: least absolute shrinkage and selection operator
ROC curve: receiver operator characteristics curve.
